# Early Onset High Myopia and Severe Anisometropia Associated With Familial Exudative Vitreoretinopathy of Irregular Dominant Inheritance in 11 Chinese Families: Analysis of Refraction Features and Pathogenic Variations

**DOI:** 10.1155/genr/9963550

**Published:** 2025-12-29

**Authors:** Wan-Yu Cheng, Wei-Ning Rong, Hui-Ping Li, Xiao-Guang Wang, Rui Qi, Xiao-Long Qi, Xun-Lun Sheng, Wei Chi

**Affiliations:** ^1^ The First Hospital of Lanzhou University, The First Clinical Medical College of Lanzhou University, No. 1 Donggang West Road Chengguan District, Lanzhou, 620102, Gansu, China, lzu.edu.cn; ^2^ Ningxia Eye Hospital, People’s Hospital of Ningxia Hui Autonomous Region, Ningxia Medical University, No 936 Huanghe East Road Jinfeng District, Yinchuan, 750001, Ningxia, China, nxmu.edu.cn; ^3^ Hubin Aier Eye Hospital, No. 1017, Huanghe 12 Road Bincheng District, Binzhou, 371602, Shandong, China; ^4^ Gansu Aier Ophthalmology and Optometry Hospital, 1228-437, Guangzhou Road Qilihe District, Lanzhou, 730050, Gansu, China; ^5^ Shenzhen Eye Hospital, Shenzhen Eye Medical Center, Southern Medical University, 18 Zetian Road Futian District, Shenzhen, 518040, Guangdong, China, fimmu.com

**Keywords:** familial exudative vitreoretinopathy, hereditary, high myopia, pathogenic, variations

## Abstract

**Purpose:**

The genetic spectrum and early clinical indicators of familial exudative vitreoretinopathy (FEVR) remain incompletely defined, and few studies have investigated the genetic variants and clinical phenotypes associated with eoHM‐FEVR and anisometropia‐FEVR patients. The purpose of this study was to screen the pathogenic variations in 11 FEVR families and analyze the refractive status and pathogenic genes in patients with irregular dominantly inherited FEVR.

**Methods:**

The patients with clinical diagnoses of eoHM‐FEVR or anisometropia‐FEVR were evaluated from October 2019 to August 2022. Comprehensive ophthalmic tests were performed on participants to confirm the phenotype. The genotype was identified using whole‐exon sequencing and further verified the results among other family members by Sanger sequencing. Normal protein structures were modeled with AlphaFold, whereas mutant variants were analyzed via PyMOL. Variant pathogenicity followed the American College of Medical Genetics and Genomics (ACMG) guidelines. The protein–protein interaction (PPI) network analysis with STRING and *k*‐means clustering was applied for detecting the interaction of genes in the candidate genes, and the ClusPro Server was used for protein–protein docking.

**Results:**

A total of 11 FEVR families were included in the study, and all the probands were found to have high myopia in both eyes or one eye before the age of 7 years. The pathogenic variants were identified in the genes *TSPAN12*, *LRP5*, and *FZD4* known to be associated with FEVR in six probands. Among 13 eoHM‐related genes, *FZD4* and *LRP2* encode proteins that can dock together as analyzed by ClusPro software.

**Conclusion:**

This study observed dominant inheritance of an irregular pattern in FEVR families, with asymmetric FEVR presenting as severe anisometropia. The eye with higher myopia often had more advanced FEVR and pronounced fundus changes. PPI network analysis revealed important modules of gene interaction, and the FZD4‐LRP2 complex protein was potentially related to high myopia development. For patients with high myopia or with obvious anisometropia in both eyes, more attention should be paid clinically to the comprehensive examination of the peripheral fundus and early genetic testing.

## 1. Introduction

Familial exudative vitreoretinopathy (FEVR; OMIM: 133780) is an inherited blinding eye disease with abnormal retinal vascular development, which was first reported by Criswick and Schepens in 1969 [1]. FEVR is a lifelong disease that typically progresses through childhood and adolescence and then settles into a quiescent state (like a dormant volcano, not an extinct volcano) after the age of 20. Some complications that seriously affect vision, such as neovascularization, vitreous hemorrhage, and retinal detachment, can occur at any age during different quiescent stages. The FEVR patients may also experience reactivation of the disease process and loss of vision in middle age [2, 3]. FEVR also shows a high degree of clinical heterogeneity [4]. In the early stage, more than half of the FEVR patients are asymptomatic and have good visual acuity because the lesions are in the peripheral part of the retina. In mild asymptomatic cases, FEVR is usually detected by eye screening after an immediate family member has been diagnosed. Therefore, its incidence has been far underestimated clinically. With the development of medical technology and genetic diagnostic technology in recent years, it has been found that the incidence of FEVR is not low in the population, and it may be hidden in the seemingly normal population, and it is one of the main causes of vision impairment in adolescents caused by hereditary eye diseases in our country and even in the world.

FEVR can be inherited in an autosomal recessive, autosomal dominant, or X‐linked trait [5, 6]. The current studies have identified 14 pathogenic genes associated with the FEVR attacks, including low‐density lipoprotein (LDL)‐related receptor‐5 (*LRP5*) (MIM 603506) [7], *FZD4* (MIM 604579) [8], *TSPAN12* (MIM 613138) [9], *ZNF408* (MIM 616454) [10], *NDP* (MIM 300658) [11], *KIF11* (MIM 148760) [12], *CTNNB1* (MIM 116806) [13], *ATOH7* (MIM 609875) [14], *RCBTB1* (MIM 607867) [15], *EVR3* (11p12–13) [16], *ILK* (MIM 602366) [17], *JAG1* (MIM 601920) [18], *CTNNA1* (MIM 116805) [19], and *CTNND1* (MIM 601045) [20].

It is called irregular dominance in some autosomal dominant diseases where and when the dominant gene of a heterozygote (Aa) does not exhibit the corresponding dominant trait for some reasons, or the transmission of the dominant trait is irregular even if the dominant trait is displayed and the degree of manifestation (condition) is different. It is used to describe a situation where the expression of the dominant allele is not consistently observed across generations or individuals, even though the allele is present, which is manifested by the complexity and diversity of genetic patterns, rather than merely a problem of penetrance [2]. This is different from incomplete penetrance, where some individuals carrying the dominant allele do not exhibit any symptoms of the disease, and the focus is on the difference between “performance and nonperformance,” without reference to the complexity of genetic patterns, and from variable expressivity, where the severity of the disease differs among individuals with the same genetic variation. The clinical manifestations of FEVR are diverse, and the patients may have asymmetric and highly variable clinical manifestations in both eyes [21, 22]. Some patients may have no obvious clinical symptoms or even peripheral retinal vascular abnormalities, but some patients may have retinal detachment, and severe cases may lead to blindness [5]. There are significant phenotypic differences in clinical manifestations between different families and even between different patients in the same family. All the above findings demonstrate that the autosomal dominant FEVR patients boast typical irregular dominance [23].

High myopia is categorized into early‐onset high myopia (eoHM) and late‐onset high myopia (loHM) based on age of onset. eoHM is defined as a refractive error ≤ −6.0 D or an axial length > 26 mm occurring in the preschool years (under 7 years of age). Anisometropia is a phenomenon in which the dioptric number of both eyes is different, and the refractive state of the two eyes is not consistent. eoHM and high anisometropia are often the earliest reasons for FEVR patients to visit the ophthalmology department. However, there are fewer analyses of genetic variants and clinical phenotypes in patients with eoHM–FEVR and anisometropia–FEVR. FEVR’s impact on retinal vascularization, eye development, and genetic factors can lead to the development of eoHM and anisometropia. The conditions are often interconnected, with FEVR contributing to refractive errors through abnormal eye growth and retinal function. Connecting phenotype and genotype is the primary step for ophthalmologists to better understand the physiological basis of the disease, as well as to offer timely therapies and to predict the prognosis [21], but there is no genotype–phenotype correlation for the ocular features observed in FEVR [24].

This paper reports on 11 FEVR patients complicated with eoHM or anisometropia, of which six patients were screened for FEVR‐related pathogenic genes by whole‐exome sequencing technology. The overall study of FEVR families with detected pathogenic genes was conducted to further analyze the refractive status and pathogenic genes in irregular dominantly inherited FEVR patients in order to provide assistance in early diagnosis of FEVR, prenatal screening, and eugenic guidance [24]. Although the genetic basis of FEVR is established, the early‐onset refractive errors as a primary phenotypic manifestation remain poorly characterized. This study addresses this gap by providing direct evidence of the strong genotype–phenotype correlation between FEVR and early, significant refractive errors, thereby establishing them as key clinical indicators for FEVR.

## 2. Methods

### 2.1. Subjects and Methods

The subjects for study were patients who were clinically diagnosed with FEVR when they visited Ningxia Eye Hospital from October 2019 to August 2022 and their family members. The probands and their guardians were aware of the study and accepted the recommendation to give consent for their one‐generation relatives (parents/siblings or children) to undergo relevant ophthalmologic examinations, on which the focus was the presence of retinal vascular dysplasia. If one or more relatives of one generation were also FEVR patients, such FEVR families were included. Complete relevant ophthalmologic examinations for the probands and all family members include manifest refraction, best‐corrected visual acuity (BCVA), slit‐lamp microscopy, indirect ophthalmoscopy, wide‐angle color fundus photography (Optos Daytona P200T), and fundus fluorescence angiography (FFA). The probands’ current medical history, past medical history, personal history, family history, and obstetrical history were obtained and recorded in detail, and the family tree was drawn. The study project was approved by the Ethics Committee of the People’s Hospital of Ningxia Hui Autonomous Region and was in strict compliance with the Declaration of Helsinki. All participants agreed to participate in this study and signed the written informed consents. In this study, dominant inheritance of irregular pattern refers to pedigrees exhibiting both incomplete penetrance, where some individuals carrying the pathogenic variant do not manifest the typical disease phenotype, and variable expressivity, where affected individuals with the same pathogenic variant show a wide spectrum of clinical severity. This assessment was based on comprehensive pedigree analysis and detailed phenotyping of all available family members.

### 2.2. Inclusion Criteria and Exclusion Criteria

The FEVR probands included in this study were consistent with the fundus change characteristic of FEVR subject to fundus examination and fluorescein fundus angiography, including peripheral retinal vascular disruption accompanied by the formation of nonperfusion areas. Exclusion criteria include history of trauma, preterm birth, oxygen inhalation, and low birth weight; hypertension, diabetes, and other systemic diseases, or combined with glaucoma, uveitis, and other retinal vascular diseases; and inability to perform fundus examination due to refractive interstitial turbidity.

### 2.3. Clinical Staging of FEVR and Definition of eoHM

Clinical staging of FEVR was performed according to the staging criteria of FEVR as specified in the literature of Pendergast SD [25]: Stage I: increased vascular branches of peripheral retina, straight vessels, arteriovenous anastomosis at the end of the vessels, fan‐shaped termination of the avascular area, no neovascularization, and no clinical symptoms; Stage II: peripheral retinal avascular area, neovascularization, sickle‐shaped retinal folds/temporal fibrovascular strips, pulling of optic disk or macula (Stage A: retinal exudation; Stage B: retinal exudation free); Stage III: limited detachment of peripheral retina without involvement of macula (Stage A: retinal exudation; Stage B: retinal exudation free); Stage IV: localized detachment of the peripheral retina involving the macula (Stage A: retinal exudation; Stage B: retinal exudation free); and Stage V: total detachment of the retina, often accompanied by loss of the anterior chamber, cataract, iris atrophy, neovascular glaucoma, and other late complications. FEVR occurs due to a congenital failure of retinal vascular development, and the disease rarely progresses. Therefore, these staging systems do not represent stages through which the disease advances [24]. eoHM: The probands themselves stated or their family members stated on behalf that the probands had high myopia in one or both eyes at preschool (< 7 years old) and were diagnosed with high myopia (equivalent spherical lens ≤ −6.00 D or axial length > 26 mm) by relevant examinations in our hospital or other hospitals, with or without any systemic abnormality in addition to the ocular abnormality. Anisometropia: It was diagnosed after a relevant examination in our hospital.

### 2.4. Next‐Generation High‐Throughput Sequencing of Whole‐Genome Exons

Next‐generation high‐throughput sequencing of whole‐genome exons was performed, and for this, 5 mL of peripheral venous blood was collected, and genomic DNA was extracted using the standard procedure of the QiAamp Blood Mini Kit DNA Extraction Kit (Qiagen, Germany). Genome‐wide exon capture was performed using the Agilent SureSelect Exon Capture Kit, and sequencing was performed using a high‐throughput sequencer (Illumina) at a depth of 100×. The original sequencing data were processed by the analysis software of Illumina base calling software version 1.7 and then compared to the Human Genome DNA Reference Sequence (NCBI Build 37.1) available with the National Center for Biotechnology Information (NCBI). Single‐nucleotide variants (SNVs) and insertion and deletion variants (indel) were analyzed using SOAP software (https://soap.genomics.org.cn) and BWA software (https://bio-bwa.sourceforge.net/), respectively, to obtain the full range of variants occurring in the DNA sequences in the samples. High‐frequency variant sites with a minimum allele frequency (MAF) > 1% in the database (db135) were screened. Variants with no effect on protein structure and function were screened. Candidate pathogenic gene variants were obtained after stepwise screening of the number of variants shared by all patients in the family, followed by screening of variants present in the family with no affected relatives. Candidate pathogenic gene variants were validated by Sanger to exclude false positives and further validated by cosegregation of genotypes and phenotypes among normal family members.

### 2.5. Pathogenicity Analysis of Variants

**Figure 1:**
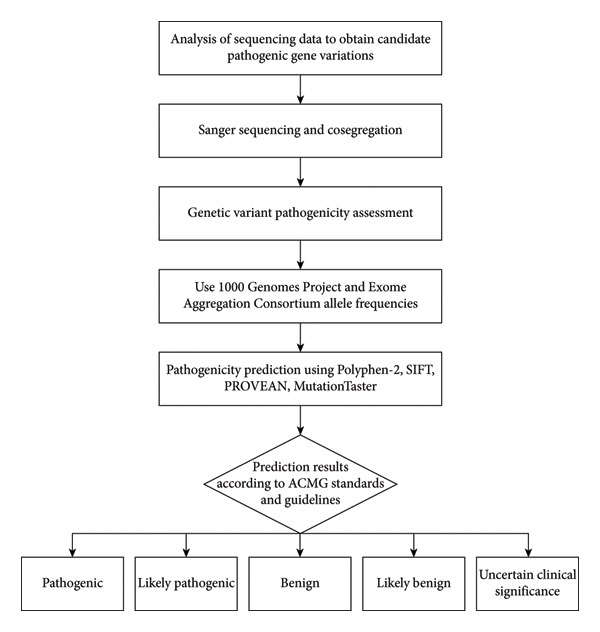
Workflow for variant pathogenicity analysis. This workflow illustrates the systematic process for analyzing and interpreting the pathogenicity of genetic variants, including variant identification, annotation, in prediction, and classification according to ACMG.

Genetic variant pathogenicity was assessed for de novo variants according to *Standards and Guidelines for Interpretation of Sequence Variants* issued by the American College of Medical Genetics and Genomics (ACMG) in 2015 [26]. MAF < 0.005 was used as the criterion to exclude benign variants by reference to the databases for East Asian populations’ allele frequencies available with the 1000 Genomes Project (1000G; https://browser.1000genomes.org) and Exome Aggregation Consortium (https://exac.broadinstitute.org/). Different prediction software packages such as Polyphen‐2 (https://genetics.bwh.harvard.edu/pph2), SIFT(https://sift.jcvi.org), PROVEAN (https://provean.jcvi.org/index.php), and MutationTaster (https://www.mutationtaster.org) were used for pathogenicity prediction. Variants were classified as of uncertain clinical significance when at least one prediction had a benign outcome or when there was insufficient evidence of pathogenicity. When all predictions were pathogenic, variants were classified as likely pathogenic in combination with other evidence. Frameshift variants, nonsense variants, and variants with experimental evidence of loss of protein function were classified as pathogenic variants. Conservation of gene sequences across species in evolution was measured using websites such as GERP++ (https://bio.tools/gerp) (see Figure 1).

### 2.6. Protein–Protein Interaction (PPI) Network Analysis

The STRING database (https://string-db.org) [27] was applied for detecting the interaction of genes, which integrated both known and predicted PPI. The confidence scores greater than 0.4 will be selected for further analysis. Functional enrichment in the network was also shown by STRING under Gene Ontology (GO) terms. In the STRING website, we get the myopia‐related gene lists under the option “Disease,” followed by a selection of options “Organism” as “Homo sapiens” paired with the term “high myopia” or “Familial Exudative Vitreoretinopathy.” After that, “SEARCH” was clicked and the original data were downloaded. Clustering of proteins was performed by *k*‐means clustering in STRING.

### 2.7. Protein–Protein Docking

The ClusPro Server (https://cluspro.org) [28] is used for protein–protein docking. The proteins in PDB format predicted by AlphaFold were downloaded from UniProt (https://www.uniprot.org) [29]. The protein structures of PDB files were uploaded to the ClusPro Server to get the docking model. The 3D structure of protein–protein docking was visualized with PyMOL.

## 3. Results

A total of 11 FEVR patients, as screened, were included in the study, and all the probands had typical manifestations of FEVR clinically after examinations. The whole‐exome sequencing identified the pathogenic genes associated with FEVR disease in six of these patients, and six probands were found to have high myopia in both eyes or one eye during childhood. All family members underwent detailed ophthalmologic examinations, and at least one patient in the family had fundus changes in FEVR besides the probands. FEVR patients in all families showed an earlier age of onset than the previous generation and a progressive worsening of the disease. Three of the probands had severe anisometropia and asymmetric fundus changes in both eyes. The higher the degree of myopia, the worse the BCVA and the more severe the degree of FEVR. The whole‐exome sequencing identified that six families involved a total of three FEVR‐related genes, including *TSPAN12*, *LRP5*, and *FZD4*.

**Figure 2:**
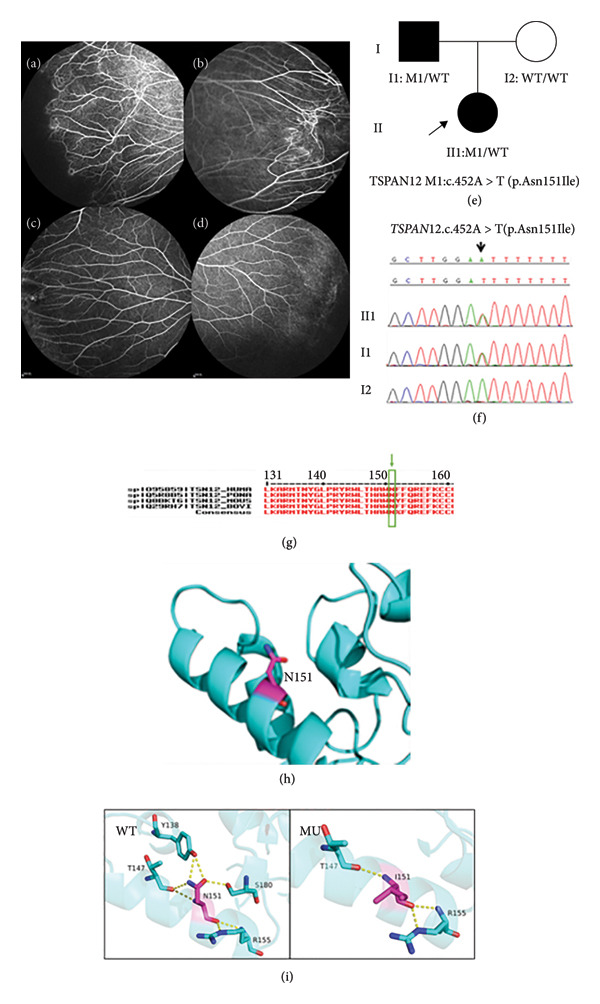
Clinical manifestations and genetic testing results in Family 1 proband. (a and b) FFA manifestations of Family 1 proband (II1): There was a distinct cristae‐like demarcation between the avascular area of the peripheral retina and the vascularized retina, and numerous branching retinal vessels were available in the shape of willow branches. The closer to the periphery, the more numerous. The terminal blood vessels became straightened. Abnormal arteriovenous anastomosis, neovascularization, and foci of subretinal exudates were observed; (c and d) FFA manifestations of the father (I1) of Family 1 proband: The terminal blood vessels became straightened, and foci of subretinal exudates were observed; (e) gene sequencing diagram (the proband had a heterozygous variant of the *TSPAN12* gene, whereas the proband’s father carried an identical heterozygous variant); (f) tree of Family 1; (g) amino acid sequence at variant site was compared with homologous protein sequences of multiple species (amino acid at variant site was highly conserved); (h) protein structure of the *TSPAN12* gene variant p.Asn151Ile; (i) wild‐type (WT) protein and p.N151I mutation (MU).

Family 1 proband, 9‐year‐old female, had BCVA: OD: 0.6 and OS: 0.16 (Table 1), and no obvious abnormality was found in the anterior segment of both eyes. The FFA test suggested that some retinal blood vessels in the fundus of both eyes had a rigid vascularization, and retinal capillary dilation, increased branching, and mild leakage in the peripheral temporal area showed the formation of patellar nonperfusion area, and abnormal retinal arteriovenous anastomosis accompanied by neovascularization was seen around the left eye (Figures 2(a) and 2(b)). Proband father, 35 years old, had BCVA: OD: 1.0 and OS: 1.0. The FFA test suggested that the capillary branches in the peripheral temporal retina of both eyes were increased, and some blood vessels were stiff. The optic disk boundaries were clear, and the peridiscal vessels were stretched and straightened (Figures 2(c) and 2(d)). This family was autosomal dominant, and a heterozygous missense variant c.452A > T(p.Asn151lle) was detected in the proband’s *TSPAN12* (Figure 2(f)), which resulted in the variation of amino acid 151 from aspartic acid to isoleucine. Such variant was not found in the normal population database and the known site database and was a newly identified variant site (PM2). All predictions of four software packages such as SIFT, PolyPhen‐2, PROVEAN, and MutationTaster are deleterious (Table 2), and such variant site was highly conserved across species (PP3) (Figure 2(g)). Sanger sequencing analysis confirmed that the father of the proband with the FEVR phenotype carried a heterozygous missense variant at the same site, and the phenotypically normal mother was wild type (WT) at such site, suggesting that the genotype and the clinical phenotype were coseparated (PP1) (Figure 2(e)). The phenotype of the variant carrier was highly consistent with the monogenic genetic disorder FEVR (PP4), and genetic testing reports from reliable reputable sources considered the variant to be pathogenic (PP5). The p.Asn151lle WT protein at Site 151 was a polar uncharged glutathione; the N atom of the side chain amide functional group formed hydrogen bonds with T147 and Y138, the O atom of the side chain amide functional group formed hydrogen bonds with S180 and Y138, and the backbone O and N atoms formed four hydrogen bonds with the proteins R155 and T147, respectively. p.Asn151lle mutation (MU) resulted in the replacement of glutathione at Site 151 by a nonpolar uncharged isoleucine, and the variant to isoleucine at Site 151 did not form hydrogen bond interactions with T147, Y138, and S180. The alteration of these amino acid interactions likely led to structural and functional alterations in the mutated protein (Figure 2(i)), suggesting that the new missense variant c.452A > T (p.Asn151lle) likely had affected the tertiary structure of the TSPAN12 protein. As stated above, this new *TSPAN12* variant likely caused disease by affecting the structure and function of the TSPAN12 protein. According to ACMG guidelines, this variant was likely pathogenic (PM2+PP4 +PP3+PP1+PP5).

**Table 1:** Clinical findings and FEVR staging in probands from six FEVR families.

Family	Proband	Age	Sex	BCVA		SE (D)		Axial length (mm)		FEVR staging	
				OD	OS	OD	OS	OD	OS	OD	OS
Family 1	Proband	9^∗^	Female	20/40	20/125	+2.375	−11.75	20.58	23.69	Stage I	Stage IIB
	Proband father	35	Male	20/20	20/20	—	—	—	—	Stage I	Stage I

Family 2	Proband	3	Male	20/200	20/50	−9.50	−1.25	25.17	22.58	Stage I	Stage I
	Proband father	41	Male	20/25	20/20	—	—	—		Stage IIIB	Stage I

Family 3	Proband	22^∗^	Female	20/80	20/80	−13.25	−14.25	26.72	27.10	Stage IIA	Stage IIA
	Proband mother	44	Female	20/20	20/20	—	—	—		Stage I	Stage I

Family 4	Proband	24^∗^	Male	20/25	20/25	−8.25	−9.13	24.98	25.20	Stage IIA	Stage IIA
	Proband mother	49	Female	20/20	20/20	—	—	—	—	Stage I	Stage I

Family 5	Proband	6	Female	20/100	20/50	−9.85	−7.33	—	—	Stage IIA	Stage IVA
	Proband father	39	Male	20/20	20/20	—	—	—	—	Stage I	Stage I

Family 6	Proband	9^∗^	Male	20/80	20/50	−11.35	−9.50	26.47	26.04	Stage IIA	Stage IIA
	Proband mother	36	Female	—	—	—	—	—	—	Stage IIA	Stage IIA

^∗^The age at which the probands were diagnosed with high myopia was all before 7 years old.

**Table 2:** Gene variant loci of six FEVR families and bioinformatic analysis results.

	Family 1	Family 2	Family 3	Family 4	Family 5	Family 6
Gene	*TSPAN12*	*TSPAN12*	*LRP5*	*FZD4*	FZD4	*FZD4*
NM_	NM_012338	NM_012338	NM_002335.4	NM_012193	NM_012193.4	NM_012193.4
Nucleotide	c.452A > T	c.543C > G	c.1330C > T	c.40_49del	c.313A > G	c.901G > T
Amino acid	p.Asn151lle	p.Cys181Trp	p.Arg444Cys	p.Pro14Ser fs^∗^43	p.Met105Val	p.Gly301^∗^
Exon	exon6	exon7	exon6	exon1	exon2	exon2
Source	Father	Father	Mother	Mother	Father	Mother
1000g2015aug_all	—	—	—	—	—	—
1000g2015aug_eas	—	—	—	—	—	—
SIFT_	D	D	D	—	T	—
Polyphen2_HDIV_	0.885	1.000	1.000	—	P	—
Polyphen2_HVAR_	0.833	1.000	0.094	—	P	—
LRT	D	D	U	—	D	—
MutationTaster	D&D	D&D	A	—	A	—
MutationAssessor	M	M	M	—	L	—
FATHMM_	T&T	D&D	D	—	T	—
PROVEAN_	D	D	D	—	N	—
CADD_	0.93945	0.82021	3.346	—	3.463	—
GERP++	0.94740	0.26032	3.94	—	5.82	—
phyloP100way_	0.75390	0.39683	2.945	—	6.271	—
phastCons100way_	0.71511	0.71511	0.99	—	1	—
REVEL_score	0.739	0.770	0.8	—	0.511	—

**Figure 3:**
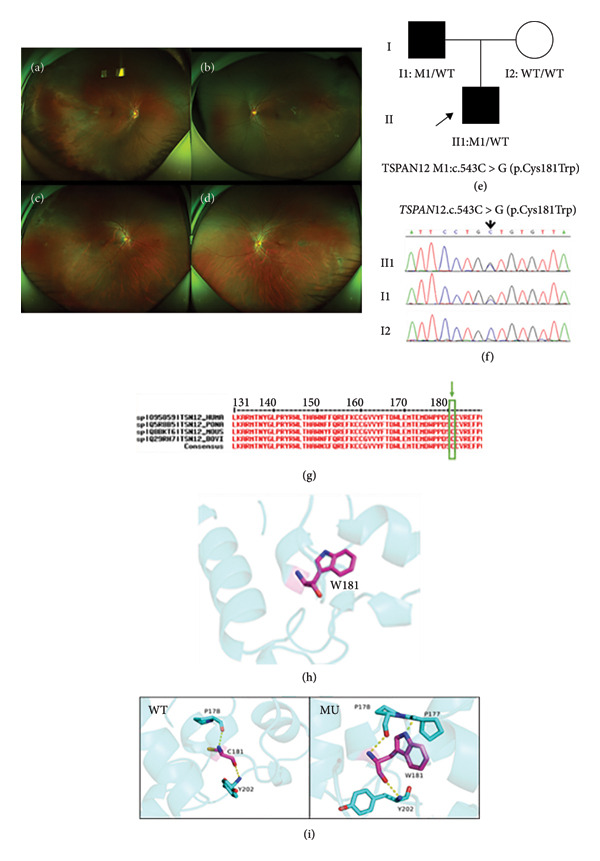
Clinical manifestations and genetic testing results in Family 2 proband. (a and b) Wide‐angle fundus photography of Family 2 proband (II1); (c and d) wide‐angle fundus photography of the father (I2) of Family 2 proband; (e) tree of Family 2; (f) gene sequencing diagram (the proband had a heterozygous variant of the *TSPAN12* gene, whereas the proband’s father carried an identical heterozygous variant); (g) amino acid sequence at variant site was compared with homologous protein sequences of multiple species (amino acid at variant site was highly conserved); (h) protein structure of the *TSPAN12* gene variant p.Cys181Trp: (i) wild‐type (WT) protein and p.C181W mutation (MU).

Family 2 proband, 3‐year‐old male, had BCVA: OD: 0.1 and OS: 0.4 (Table 1), and no obvious abnormality was found in the anterior segment of both eyes. Fundus photography showed straightening of peripheral retinal vascularization in both eyes (Figures 3(a) and 3(b)). The affected father, 41 years old, had BCVA: OD: 0.8 and OS: 1.0. Fundus photography showed straightening of peripheral retinal vascularization in both eyes and lattice degeneration of the peripheral retina (Figures 3(c) and 3(d)). The family was autosomal dominant, and a heterozygous missense variant c.543C > G (p.Cys181Trp) was detected in the *TSPAN12* of the proband, which resulted in a variation of amino acid at Site 181 from cysteine to tryptophan. Such variant was not found in the normal population database and the known site database and was a newly identified variant site (PM2). All predictions of software packages such as SIFT, PolyPhen‐2, MutationTaster, and FATHMM are deleterious (Table 2), and such variant site was highly conserved across species (Figure 3(g)) (PP3). Sanger sequencing analysis confirmed that the father of the proband with the FEVR phenotype carried a heterozygous variant at the same site, and the phenotypically normal mother was WT at such site, suggesting that the genotype and the clinical phenotype were coseparated (PP1) (Figures 3(e) and 3(f)). The phenotype of the variant carrier was highly consistent with the monogenic genetic disorder FEVR (PP4), and genetic testing reports from reliable reputable sources considered the variant to be pathogenic (PP5). PyMOL was applied to produce 3D structural images of proteins (Figure 3(h)). The p.Cys181Trp WT protein was a polar uncharged cysteine at Site 151; backbone O and N atoms formed two hydrogen bonds with proteins P178 and Y202, respectively, p.Cys181Trp MU resulted in the replacement of the cysteine at Site 181 by a polar uncharged tryptophan, and the N atom of the indole backbone mutated to an isoleucine amino acid at Site 181 formed a hydrogen‐bonding interaction with P177. Alterations in these amino acid interactions likely resulted in altered protein structure and function after variation (Figure 3(i)). As stated above, this new *TSPAN12* variant likely caused disease by affecting the structure and function of the TSPAN12 protein. According to ACMG guidelines, this variant was likely pathogenic (PM2 + PP4 + PP3 + PP1 + PP5).

**Figure 4:**
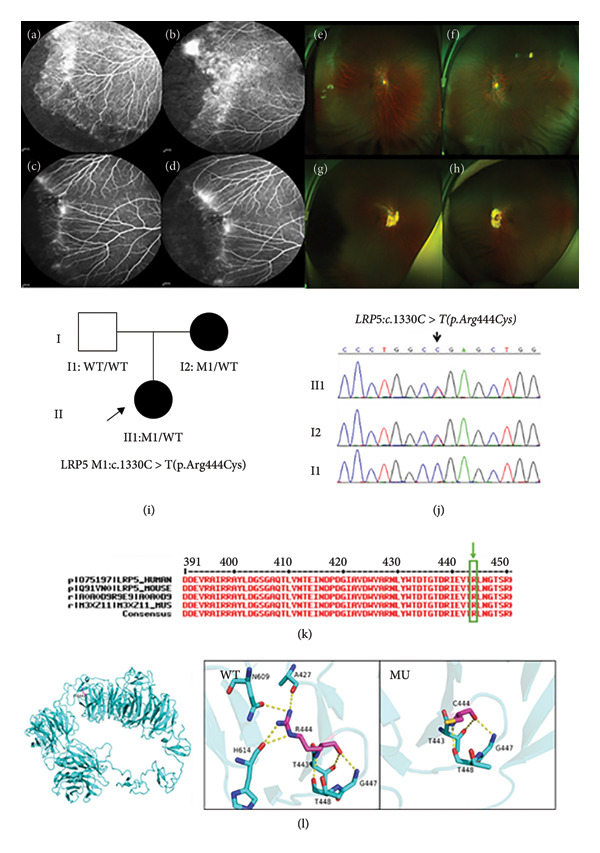
Clinical manifestations and genetic testing results in Family 3 proband. (a and b) FFA manifestations of Family 3 proband (II1): There was a distinct cristae‐like demarcation between the avascular area of the peripheral retina and the vascularized retina, and numerous branching retinal vessels were available in the shape of willow branches. The closer to the periphery, the more numerous. The terminal blood vessels became straightened. Abnormal arteriovenous anastomosis, neovascularization, and foci of subretinal exudates were observed; (c and d) FFA manifestations of the mother (I2) of Family 3 proband: The terminal blood vessels became straightened, and foci of subretinal exudates were observed; (e and f) wide‐angle fundus photography of Family 3 proband (II1); (g and h) wide‐angle fundus photography of the mother (I2) of Family 3 proband; (i) tree of Family 3; (j) gene sequencing diagram (the proband had a heterozygous variant of the *LRP5* gene, whereas the proband’s mother carried an identical heterozygous variant); (k) amino acid sequence at variant site was compared with homologous protein sequences of multiple species (amino acid at variant site was highly conserved); (l) protein structure of the *LRP5* gene variant p.Arg444Cys: wild‐type (WT) protein and p.R444C mutation (MU).

Family 3 proband, 22‐year‐old female, had BCVA: OD: 0.25 and OS: 0.25 (Table 1), and no obvious abnormality was found in the anterior segment of both eyes. Fundus photography showed straightening of peripheral retinal vascularization in both eyes (Figures 4(e) and 4(f)); the FFA test suggested that the peripheral retina of both eyes showed a cristae‐like demarcation between the avascular area and the vascularized retina, with numerous branching retinal vessels in the shape of willow branches and anomalous arteriovenous anastomoses accompanied by neovascularization (Figures 4(a) and 4(b)). The affected mother, 44 years old, had BCVA: OD: 1.0 and OS: 1.0, and no obvious abnormality was found in the anterior segment of both eyes. Fundus photography showed parapapillary atrophy visible around the optic disks and straightening of peripheral retinal vascularization in both eyes (Figures 4(g) and 4(h)). The FFA test suggested that the vascularization of the retinal endings became straightened in both eyes, and the capillaries showed brush‐like changes with mild leakage (Figures 4(c) and 4(d)). The family was autosomal dominant, and a heterozygous variant c.1330C > T(p.Arg444Cys) was detected in the *LRP5* of the proband (Figure 4(j)), which resulted in a variation of amino acid at Site 444 from arginine to cysteine. Such variant was not found in the normal population database and the known site database and was a newly identified variant site (PM2) (Table 2). All predictions of software packages such as SIFT, PolyPhen‐2, MutationTaster, FATHMM, and PROVEAN are deleterious (Table 2), and such variant site was highly conserved across species (Figure 4(k)) (PP3). Sanger sequencing analysis confirmed that the mother of the proband with the FEVR phenotype carried a heterozygous variant at the same site, and the phenotypically normal father was WT at such site, suggesting that the genotype and the clinical phenotype were coseparated (Figure 4(i)) (PP1). In vitro functional experiments demonstrated that this variant resulted in a 45% decrease in Norrin signaling transduction activity (PS3), and the phenotype of variant carriers was highly consistent with the monogenic genetic disorder FEVR (PP4). PyMOL was applied to produce 3D structural images of proteins. The p.Arg444Cys WT protein was a polar positively charged arginine at Site 444, the N atom of the functional group of its side chain guanidine formed hydrogen bonds with A427, N609, and H614, and the backbone O and N atoms formed triple hydrogen bonds with proteins G447, T448, and T443, respectively, p.Arg444Cys MU resulted in the replacement of the arginine at Site 444 by the polar uncharged cysteine, and the variation to the 444 cysteine at Site 444 did not allow for hydrogen‐bonding interactions with A427, N609, and H614, and alterations in these amino acid interactions likely resulted in altered protein structure and function after variation (Figure 4(l)). As stated above, this new *LRP5* variant likely caused disease by affecting the structure and function of the LRP5 protein. According to ACMG guidelines, this variant was likely pathogenic (PS3 + PM2 + PP1 + PP3 ++ PP4).

**Figure 5:**
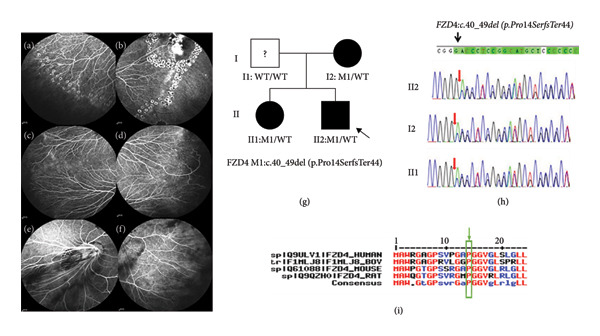
Clinical manifestations and genetic testing results in Family 4 proband. (a and b) FFA manifestations of Family 4 proband (II2): There was a distinct cristae‐like demarcation between the avascular area of the peripheral retina and the vascularized retina; numerous branching retinal vessels were available in the shape of willow branches. The closer to the periphery, the more numerous. The terminal blood vessels became straightened. Abnormal arteriovenous anastomosis, neovascularization, and foci of subretinal exudates were observed, and a large number of old laser speckles are visible in the peripheral retina and around the exudative lesions; (c and d) wide‐angle fundus photography of the mother (I2) of Family 4 proband: There were many branches available with the peripheral retinal vessels in the shape of willow branches. The closer to the periphery, the more numerous. The terminal blood vessels became straightened. (e and f) FFA manifestations of the sister of Family 4 proband (II 1): The blood vessels in the temporal side of the optic disk became straight and pulled toward the temporal side; there was an increase in the branching of the temporal peripheral veins and a straightening of the blood vessels; (g) tree of Family 4; (h) gene sequencing diagram (the proband had a frameshift variant of the *FZD4* gene, whereas the proband’s mother and sister carried an identical variant); (i) amino acid sequence at variant site was compared with homologous protein sequences of multiple species (amino acid at variant site was highly conserved).

Family 4 proband, 24‐year‐old male, had BCVA: OD: 0.8 and OS: 0.8 (Table 1), and no obvious abnormality was found in the anterior segment of both eyes. The FFA test suggested that the peripheral retina of both eyes showed a cristae‐like demarcation between the avascular area and the vascularized retina, neovascularization accompanied by fluorescein leakage, and a large number of old laser speckles visible in the peripheral retina (Figures 5(a) and 5(b)). The affected mother is 49 years old. The FFA test suggested that numerous branching retinal vessels were available in the shape of willow branches, and the vascularization of the retinal endings became straightened in both eyes (Figures 5(c) and 5(d)). The affected sister is 28 years old. The FFA test suggested that the blood vessels in the temporal side of the optic disk of the right eye became straight and pulled toward the temporal side, and there was an increase in the branching of the temporal peripheral veins and avascularity of the bilateral peripheral retina (Figures 5(e) and 5(f)). The family was autosomal dominant, and a heterozygous frameshift deletion variant c.40_49del(p.Pro14SerfsTer44) was detected in the *FZD4* of the proband (Figure 5(g)). The variant was a base deletion at Sites 40–49, which resulted in the variation of amino acid at Site 14 from proline to serine and the occurrence of premature translational termination codon at the next 44 amino acids. Such variant was not found in the normal population database and the known site database and was a newly identified variant site (PM2) (Table 2). The occurrence of frameshift variation followed by nonsense variations resulted in the absence of transcripts or degradation of transcripts caused by nonsense variations, causing complete loss of gene products and disruption of gene function. The frameshift deletion variant c.797_801del (p.Val266Alafs^∗^75) was pathogenically very strong (PVS1) according to ACMG guidelines for the classification of pathogenic variants, and such site was highly conserved (PP1) (Figure 5(i)) subject to analysis for conservativeness of amino acid sequence. Sanger sequencing analysis confirmed that the mother and sister of the proband with the FEVR phenotype carried a heterozygous variant at the same site, suggesting that the genotype and the clinical phenotype were coseparated (PP1) (Figure 5(g)). According to the standards and guidelines for the interpretation and evaluation of sequence variants, this variant was pathogenic (PVS1+PM2+PP3+PP1).

**Figure 6:**
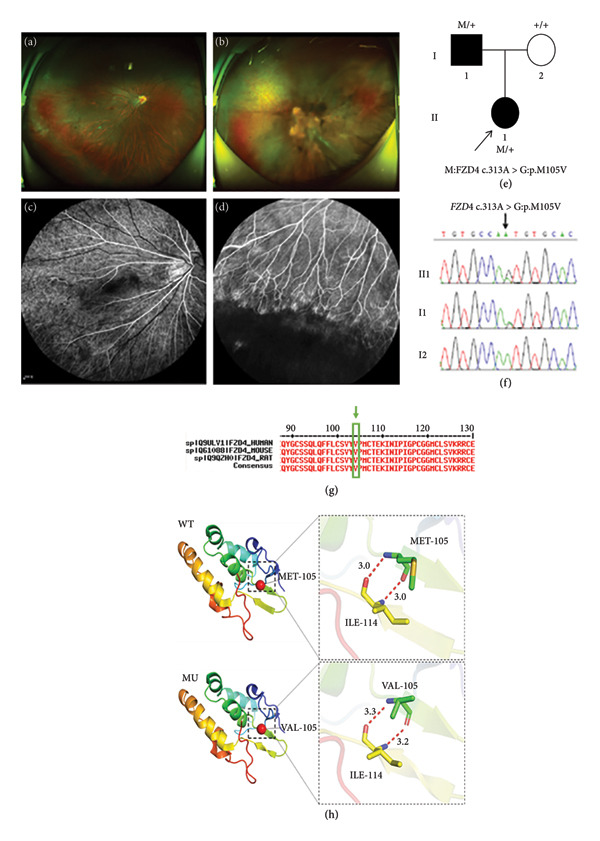
Clinical manifestations and genetic testing results in Family 5 proband. (a) Wide‐angle fundus photography of the right eye in proband (II2): The right eye had a tessellated retina in the fundus, and the fundus blood vessels were straight and pulled toward the temporal side; (b) wide‐angle fundus photography of the proband (II2): The left eye had a retinal detachment with poorly defined fundus details; (c and d) wide‐angle fundus photography of the right eye of the proband (II2): The retinal vessels were undeveloped to the periphery and terminated before the serrated rim, the avascular area and normal vascularization formed a distinct cristae‐like demarcation, and there were many branches available with the peripheral retinal vessels, which were changed in a brush‐like manner and showed a small amount of leakage; (e) family tree; (f) family members I1 and II1 carried the *FZD4* gene c.313A > G(p.M105V) heterozygous missense variant; (g) amino acid sequence at variant site was compared with homologous protein sequences of multiple species (amino acid at variant site was highly conserved); (h) protein structure of the *FZD4* gene variant p.Met105Val: wild‐type (WT) protein and p.Met105Val mutation (MU).

Family 5 proband, 6‐year‐old female, had BCVA: OD: 0.2 and OS: 0.4 (Table 1). BCVA reexamined 6 months later had OD: 0.5 and OS: manual. No obvious abnormality was found in the anterior segment of both eyes. Fundus photography showed that the right eye had a tessellated retina, the fundus blood vessels were straight and pulled toward the temporal side (Figure 6(a)), and the left eye had a retinal detachment with poorly defined fundus details (Figure 6(b)). The FFA test suggested that the retinal vessels in the right eye were undeveloped to the periphery and terminated before the serrated rim, the avascular area and normal vascularization formed a distinct cristae‐like demarcation, and there were many branches available with the peripheral retinal vessels, which were changed in a brush‐like manner and showed a small amount of leakage (Figures 6(c) and 6(d)). The affected father had mild myopia in both eyes. The FFA test suggested that there were increased branches available with the peripheral retinal vessels, and the terminal vessels were abnormally anastomosed. This family was autosomal dominant, and a heterozygous missense variant c.313A > G(p.Met105Val) was detected in the proband’s *FZD4* (Figure 6(f)), which resulted in the variation of the amino acid at Site 105 from methionine to valine. Such variant was not found in the normal population database and the known site database and was a newly identified variant site (PM2). All predictions of four software packages such as SIFT, PolyPhen‐2, PROVEAN, and MutationTaster are deleterious (Table 2), and such variant site was highly conserved across species (Figure 6(g)) (PP3). Sanger sequencing analysis confirmed that the father of the proband with the FEVR phenotype carried a heterozygous missense variant at the same site, and the phenotypically normal mother was WT at such site, suggesting that the genotype and the clinical phenotype were coseparated (Figure 6(e)) (PP1), and genetic testing reports from reliable reputable sources considered the variant to be pathogenic (PP5). PyMOL was applied to produce a 3D structural image of the protein. At Site 105 of the WT FZD4 protein, the N and O atoms of the backbone of nonpolar uncharged methionine formed hydrogen bonds with the O and N atoms formed by the nonpolar uncharged valine at Site 114, and such hydrogen bonds were at distances of 3.0 and 3.0A. The p.Met105Val variation resulted in the replacement of the uncharged methionine at Site 105 by a nonpolar uncharged valine, and the N and O atoms of the main chain of the nonpolar uncharged valine at Site 105 formed hydrogen bonds with the O and N atoms of the nonpolar uncharged valine at Site 114 at distances of 3.3 and 3.2 A (Figure 6(h)). The alterations in the interaction of these amino acids likely resulted in the alteration of the structure and function of the protein after the variation. As stated above, this new *FZD4* variant likely caused disease by affecting the structure and function of the FZD4 protein. According to ACMG guidelines, this variant was likely pathogenic (PS3 + PM2 + PP1 + PP3 ++ PP4).

**Figure 7:**
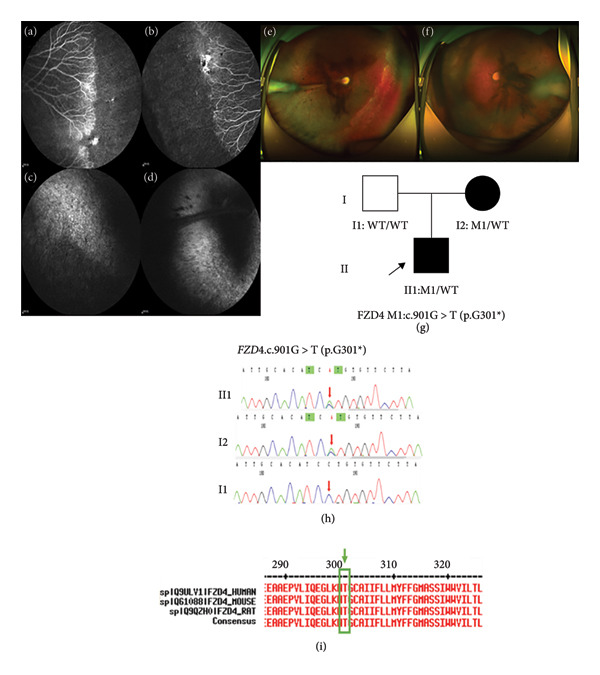
Clinical manifestations and genetic testing results in Family 6 proband. (a and b) FFA manifestations of proband (II2): The retinal vessels were undeveloped to the periphery and terminated before the serrated rim, the avascular area and normal vascularization formed a distinct cristae‐like demarcation, and there were many branches available with the peripheral retinal vessels, which were changed in a brush‐like manner and showed a small amount of leakage; (c and d) FFA manifestations of the proband’s mother (I2): The white‐striped vascular bundles above the temporal part of the optic disk extended from the optic disk to the periphery, and retinal vessels were undeveloped to the periphery, with anteriorly clear details; (e and f) wide‐angle fundus photography of the proband’s mother (I2); (g) tree of Family 6; (h) family members I2 and II1 carried the *FZD4* gene: c.901G > T (p.G301^∗^) heterozygous nonsense variant; (i) amino acid sequence at variant site was compared with homologous protein sequences of multiple species (amino acid at variant site was highly conserved).

Family 6 proband, 9‐year‐old male, had BCVA: OD: 0.25 and OS: 0.4 (Table 1). No obvious abnormality was found in the anterior segment of both eyes. Fundus photography showed that both eyes had a tessellated retina, and the peripheral retinal vessels were straight. The FFA test suggested that the retinal vessels in both eyes were undeveloped to the periphery and terminated before the serrated rim, the avascular area and normal vascularization formed a distinct cristae‐like demarcation, and there were many branches available with the peripheral retinal vessels, which were changed in a brush‐like manner and showed a small amount of leakage (Figures 7(a) and 7(b)). The affected mother had poor eyesight in both eyes since childhood. Fundus photography in both eyes showed the tessellated retina, the white‐striped vascular bundles in the temporal side of the optic disk extended from the optic disk to the periphery in the right eye, and the peripheral retinal blood vessels in the temporal side went stiff. The peripheral retina of the left eye was poorly detailed (Figures 7(e) and 7(f)). The FFA test suggested that the white‐striped vascular bundles above the temporal part of the optic disk extended from the optic disk to the periphery in both eyes, and retinal vessels were undeveloped to the periphery, with anteriorly clear details (Figures 7(c) and 7(d)). This family was autosomal dominant, and a heterozygous missense variant c.901G > T(p.Gly301^∗^) was detected in the proband’s *FZD4* (Figure 7(h)). Such variant was a change in the cDNA from base G to base T at Site 901, resulting in the codon at Site 301 changing from encoding glycine to termination codon. The mechanism of *FZD4* pathogenesis was loss of function (LOF), and this variant was nonsense and likely led to LOF of protein (PVS1). This variant was rare and not included in the gnomAD database for the East Asian general population (PM2). Sanger sequencing analysis confirmed that the mother of the proband with the FEVR phenotype carried a heterozygous nonsense variant at the same site, and the phenotypically normal father was WT at such site, suggesting that the genotype and the clinical phenotype were coseparated (PP1) (Figure 7(g)). Such site was highly conserved (PP1) (Figure 7(i)) subject to analysis for conservativeness of amino acid sequence. According to the standards and guidelines for the interpretation and evaluation of sequence variants, this variant was pathogenic (PVS1 + PM2 + PP1).

**Figure 8:**
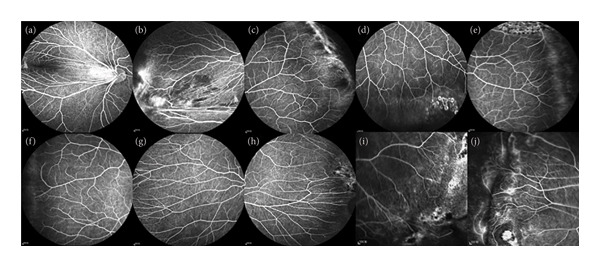
Clinical manifestations of patients with negative genetic testing results. (a and b) FFA manifestations of Proband 1 (II1): The white‐striped vascular bundles above the temporal part of the optic disk extended from the optic disk to the periphery, and retinal vessels were undeveloped to the periphery, the terminal blood vessels became straightened, and a small amount of leakage was visible; (c and d) FFA manifestations of the proband 2: The retinal vessels were undeveloped to the periphery, and there were many branches available with the peripheral retinal vessels, which were changed in a brush‐like manner and showed a small amount of leakage; (e and f) FFA manifestations of the Proband 3: The retinal vessels were undeveloped to the periphery, there was a distinct cristae‐like demarcation between the avascular area and the vascularized retina, and there were many branches available with the peripheral retinal vessels, which were changed in a brush‐like manner; (g and h) FFA manifestations of Proband 4: Terminal retinal vessels were available in the shape of willow branches. The closer to the periphery, the more numerous. A small amount of leakage was available with the terminal blood vessels; (i and j) FFA manifestations of the Proband 5: The retinal vessels were undeveloped to the periphery, there were many branches available with the peripheral retinal vessels, and a small amount of leakage was available with the terminal blood vessels.

The clinical symptoms of the other four FEVR patients were consistent with the changes in FEVR after detailed ophthalmologic examination (Figure 8), but no disease‐related pathogenic genes were detected by whole‐exome sequencing. The negative genetic results in these families highlight the complexity of FEVR and suggest the possibility of undiscovered genetic factors, such as rare or novel genes, epigenetic modifications, variants in noncoding regions, or large structural variants [30].

*TSPAN12* encodes a transmembrane protein involved in the Norrin receptor complex. Functional studies have shown that variants can impair Norrin binding or receptor complex formation, reducing downstream activation of the Wnt/β‐catenin pathway, which is essential for proper retinal vascular development [9]. *LRP5* is an LDL receptor‐related protein that interacts with Norrin and *FZD4* to activate the Wnt signaling cascade [6]. Variants in *LRP5* can affect its ability to bind Norrin, leading to a decrease in *β*‐catenin activation and the subsequent disruption of retinal angiogenesis [7]. *FZD4* is a receptor in the frizzled family, and *FZD4* works with *LRP5* and Norrin to transduce Wnt signaling. Variants in *FZD4* can hinder the receptor’s ability to transduce Norrin signals, leading to similar disruptions in the retinal vasculature [31, 32].

**Figure 9:**
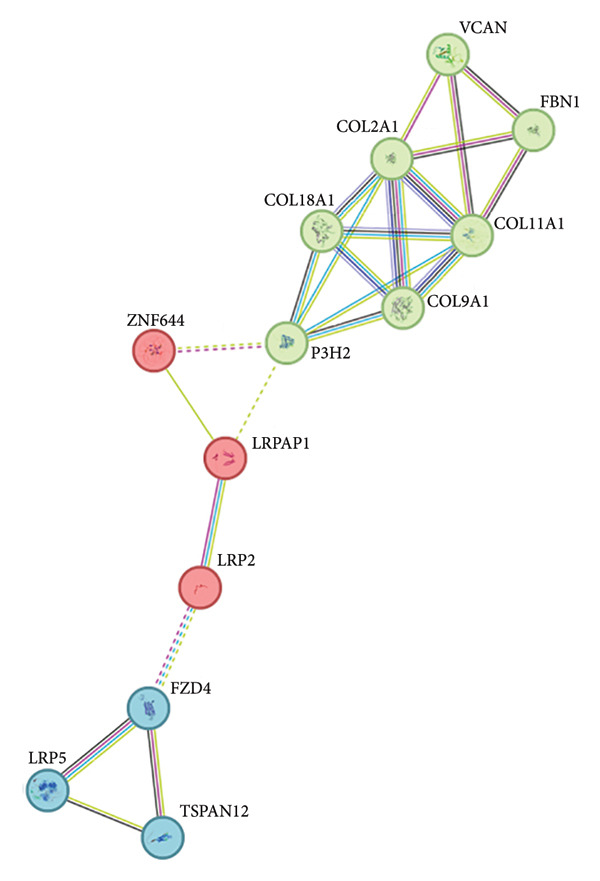
The cluster analysis by the STRING website. The green node represents Cluster 1 collagen‐containing extracellular matrix cellular components, red nodes belong to endosome lumen components, and the blue node is involved in the Wnt signaling pathway. The different colored nodes represent different groups that are connected by dashed lines.

**Figure 10:**
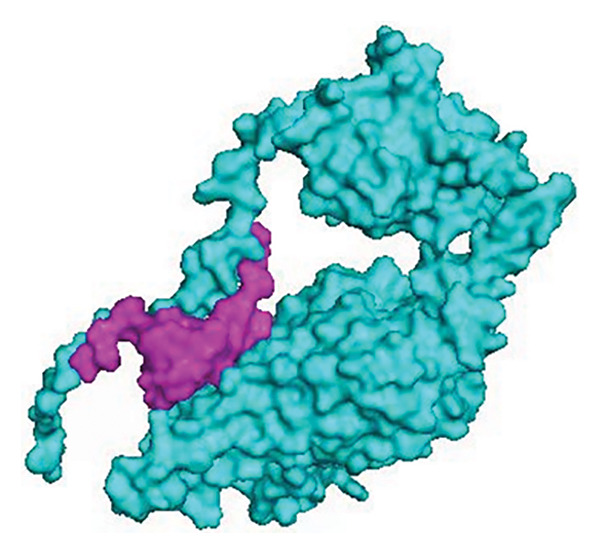
The docking of *FZD4* and *LRP2* by ClusPro software. Magenta represents the FZD4 protein, and blue represents the LRP2 protein.

The main output of the STRING database is a diagram of the protein interaction network. The nodes of these diagrams represent proteins, and the edges represent interactions between proteins. In addition, the strength of the protein interaction can also be indicated by the linear dimension and the linear width. The lines between the nodes represent the interaction between the two proteins, and different colors correspond to different types of interactions. In our study, the PPI network comprised 13 nodes with an average node degree of 3.54; the PPI enrichment *p*‐value was < 1.0e − 16, and the local clustering coefficient was 0.838. The GO analysis revealed that all 13 candidate genes in one module were associated with two molecular functions such as extracellular matrix (ECM) structural constituent and glycosaminoglycan binding with an FDR *p*‐value < 0.0001. Cluster analysis shows these 13 eoHM‐related genes belong to three clusters. Cluster 1 belongs to the collagen‐containing ECM cellular component including *COL11A1, COL18A1, COL2A1, COL9A1, P3H2,* and *FBN1* genes; Cluster 2 belongs to endosome lumen including *LRP2, LRPAP1,* and *ZNF644* genes; and Cluster 3 belongs to the Wnt signaling pathway including *LRP5, TSPAN12,* and *FZD4* genes. The results of the cluster analysis were presented in a graph (Figure 9), in which different groups are represented by different colored nodes that are connected by dashed lines. Blue node is involved in Wnt signaling pathway, and there is more than one line between the two proteins, which indicates that there are multiple interactions between the two proteins. Among the 13 eoHM‐related genes, *FZD4* and *LRP2* encode proteins that can dock together as analyzed by ClusPro software. The complex protein FZD4–LRP2 plays a bridge role among eoHM‐related genes according to PPI network analysis (Figures 9 and 10).

## 4. Discussion

FEVR patients have a variety of clinical phenotypes. In mild cases, peripheral retinal changes do not cause any symptoms. In moderate‐to‐severe cases, preretinal neovascularization and fibrosis at the junction between vascular and avascular retina can occur that causes traction of the macula and retinal vessels, where it is not even possible to recognize the characteristic manifestations of FEVR [33–35]. FEVR may be combined with or misdiagnosed as a variety of other ocular diseases such as macular laminar holes, macular holes, chorioretinopathy, macular telangiectasia Type 1 or Coats’ disease, and *persistent* hyperplastic primary vitreous (PHPV). Currently, the clinical diagnosis of FEVR is based on typical clinical manifestations, fundus examination, and FFA. The reason that some children and adolescents with FEVR first seek ophthalmology is that they have different degrees of myopia, high myopia, or severe anisometropia in both eyes. When there is no retinal detachment, clinicians focus more on the refractive changes in the patients and thus may neglect the examination of the peripheral fundus. Therefore, misdiagnosis and underdiagnosis of FEVR are clinically more common [36]. With advances in genetics and molecular biology, more and more pathogenic genes have been confirmed to be related to the pathogenesis of FEVR. Therefore, it is necessary to identify the genetic etiology and obtain an early and accurate diagnosis in patients with high myopia or severe anisometropia in both eyes, especially those without clinical symptoms and presenting only with peripheral retinal involvement.

FEVR has two typical clinical features as disease asymmetry (varying severity between eyes) and intrafamilial variability (individuals with the same variation site in the same family show different severity of the disease) [24, 37]. Natung T [22] reported two cases of FEVR from northeast India, highlighting the asymmetrical presentation of both eyes in FEVR patients. Both FEVR patients had poor visual acuity in one eye since birth, and fundus examination and FFA showed abnormal retinal vascular development in that eye. Chen C [38] found that among 621 patients clinically diagnosed with FEVR, 20 patients showed obvious binocular asymmetry in clinical manifestations, and the most common clinical manifestations of unilateral severe eyes were total retinal detachment (12 cases, 60%) and retinal folds (6 cases, 30%). Genetic testing results showed that the *LRP5* variant was the most common (11 cases, 55%).

It was found from the study that the more myopic the patient was, the more severe the degree of fundus lesion in FEVR was. This might be a distinct form caused by phenotypic heterogeneity and nonpenetrance. Patients with unilaterally significant abnormalities of FEVR may be clinically underdiagnosed or misdiagnosed in the disguise of other vitreoretinal disorders, such as persistent fetal vasculature syndrome (PFVS) [23]. Therefore, clinicians are kindly advised to conduct further FFA tests for monocular FEVR patients to confirm the diagnosis. FEVR is highly clinically heterogeneous [24, 37], as some patients only show increased peripheral retinal vascular branches, straight vessels, and no significant vision loss, and it is sometimes difficult to make a definitive early diagnosis of FEVR if patients are treated as isolated individuals.

Furthermore, it was noted in this study that FEVR probands in all families showed an earlier age of onset than the previous generation and more severe fundus abnormalities in the fundus examination and fluorescein angiography. Dominant inheritance of irregular pattern refers to a pattern of inheritance in which the traits associated with a particular gene are not expressed in every generation, and the expression of the traits can vary in a manner that is not strictly consistent with the typical dominant inheritance pattern. In cases of irregular dominant inheritance, there may be factors that modify the expression of the trait, such as modifier genes, environmental factors, or variable penetrance. The clinical manifestations and genetic analysis of these cases in our study are consistent with the characteristics of irregular dominant inheritance. Kashani [37] reported a study on the prevalence and severity of FEVR in asymptomatic relatives and found that only 8% of first‐degree relatives of patients had a positive history of FEVR. Of the asymptomatic family members screened, 58% had clinical and angiographic manifestations of FEVR Stage I or II, and 21% had clinical or angiographic manifestations of Stage III or above. Findings suggest that mild asymptomatic FEVR may be hidden in the seemingly normal population. Therefore, family members of patients who have been diagnosed with FEVR should undergo the FFA test and genetic testing, which may allow early detection of mild or asymptomatic FEVR. It is beneficial for clinicians to actively assess and intervene to mitigate the potential risk of the patients. A holistic study of FEVR families not only helps to make an accurate and early diagnosis but also contributes to a better understanding of the pathogenesis of FEVR.

In the present study, a total of three pathogenic genetic variants were identified in six FEVR families whose pathogenic genes were detected by whole‐exon gene sequencing. The previous studies confirmed that variation in six genes (*FZD4*, *TSPAN12*, *NDP*, *LRP5*, *CTNNB1*, and *CTNNA1*) associated with the Norrin/Wnt signaling pathway explained 50% of FEVR cases [33]. This pathway is highly conserved in biological evolution and plays an important role in retinal angiogenesis [38]. Among these pathogenic genes, *FZD4*, *LRP5*, and *TSPAN12* are the most common pathogenic genes [32]. In our study, five of 11 families had no identified pathogenic variants. Given the unique phenotype, we hypothesize that these families might carry variants in new genes interacting with known pathways. The unresolved cases may reflect limitations of current methods to identify novel genetic mechanisms requiring further investigation. In addition, nongenetic factors including environmental influences such as diet, toxins, or prenatal conditions may also contribute to FEVR. Other possibilities include somatic mosaicism, where variations in specific cells may not be detected in genetic testing, incomplete penetrance of certain variants, and multifactorial inheritance, where multiple genetic and environmental factors interact to increase disease susceptibility. These factors suggest that a single genetic variant may not fully explain the disease in all cases. Understanding the full spectrum of genetic and environmental contributions to FEVR will be crucial for improving diagnosis, treatment, and prevention strategies [26].

According to the PPI network analysis and protein docking model, the protein of versican interacts with fibrillin‐1, which is the component of ECM; the versican and fibrillin‐1 complex links extracellular microfibrils to other connective tissue networks and may be responsible for high myopia [39–41]. *FZD4* is another key factor activating the Wnt pathway, which contributes to myopia. Xu confirmed Fzd4−/− knockout mice display congenital retinal hypovascularization similar to that observed in FEVR, defining a Norrin‐Fz4 signaling system that plays a central role in vascular development in the eye and ear [42, 43]. A 2025 study identified five novel pathogenic *FZD4* variants, and all these variants significantly reduce *β*‐catenin signaling activity in dual‐luciferase reporter assays compared to the WT protein. This functional evidence is crucial for confirming the pathogenic mechanism of new genetic variants [44]. *LRP5*, a member of the LDL receptor superfamily, is a coreceptor for Wnt ligands involved in the wingless (Wnt) signaling pathway, which is essential for the development of vascular endothelial cells, Müller cells, and retinal interneurons, which is closely related to high myopia [45, 46]. As we know, *LRP2* variants can cause Donnai–Barrow syndrome (DBS) and high myopia is a major feature of DBS. Studies in mammals demonstrate *LRP2* expression in the ciliary epithelium, retinal pigment epithelium layer, and retinal neurosensory layer of the eye, which is critical for normal ocular development and function [47]. Besides, other animal studies also showed that the *LRP2*‐deficient eyes were represented with increased eye lengthening by postnatal Day 5 and it was accompanied by a rapid decrease in the bipolar, photoreceptor, and retinal ganglion cells, and eventually the optic nerve axons, indicating that the function of *LRP2* in the ocular tissues is necessary for normal eye growth. Moreover, the *LRP2* knockout mice show severe high myopia [48]. PPIs can help find key proteins that play important roles in biological processes, as well as identify important biological processes and pathways. In analyzing complex biological processes and pathways, protein interactions can be better understood and proteins and pathways of potential importance can be discovered [47, 48]. In the pathogenicity of FEVR, the FZD4‐LRP5 and FZD4‐NDP interactions play significant roles in the Wnt signaling pathway, including cell fate determination, cell proliferation, and organ development. In this study, PPI network analysis showed the complex protein FZD4‐LRP2 plays a bridge role among eoHM‐related genes. It implied that when the *FZD4* gene is inactivated as a variant, the function of the LRP2 protein will be affected, which may be potentially responsible for high myopia. However, further studies would be needed to pinpoint the exact cell types within the retina (e.g., photoreceptors, ganglion cells, and Muller cells) where the interaction takes place. The interaction between *FZD4* and *LRP2* is crucial for retinal vascular development through Wnt/β‐catenin signaling. *LRP2*, a large endocytic receptor, may act as a coreceptor with *FZD4*, modulating the pathway in the retina [49].

As an inherited eye disease, genetic testing of the FEVR family as an integral whole in combination with clinical examination can be beneficial to an early and accurate diagnosis of the disease, and timely identification of FEVR patients without special clinical symptoms shall be contributive to early intervention and treatment, and complications such as retinal detachment that further affect the vision could be avoided accordingly. Furthermore, lack of functional validation (e.g., in vitro or in vivo studies) limits the strength of the conclusions, specifically the use of cell‐based models and mouse models to assess the functional impact of these variants on retinal development, vascularization, and refractive status. This will help to provide more direct evidence for the role of these genetic variants in FEVR and strengthen the conclusions of the study.

As a retrospective study, the following limitations are available in addition to the inherent defects of retrospective clinical studies. First, our conclusions are drawn from a relatively small cohort of 11 families. Although this deeply phenotyped cohort was instrumental in identifying a novel genotype–phenotype correlation, the small sample size may limit the statistical power of our analysis and the generalizability of our findings to broader populations. Future studies with larger, multicenter cohorts are warranted to validate and extend our observations. Second, fundus fluorescein angiography is essential for the diagnosis of FEVR, which allows early identification of the disease for timely treatment and also helps to detect early asymptomatic patients. However, the images were not available or were of poor quality because the probands were too young to cooperate with the examination. Finally, refractive errors are known to be influenced by a combination of genetic and environmental factors. Although our genetic findings are robust within these families, we were unable to systematically collect or adjust for these potential environmental confounders. Therefore, future investigations that incorporate detailed environmental exposure assessments will provide a more holistic understanding of the determinants of refractive outcomes in FEVR.

High myopia and anisometropia may be clinically the earliest reason for children to visit the doctors and an important clue for the clinician in detecting underlying ocular disease. For patients with high myopia or severe anisometropia, more attention should be paid to the comprehensive examination of the peripheral fundus and genetic testing. Genetic testing can assist clinical diagnosis, improve diagnostic accuracy, determine the mode of inheritance, and provide a basis for genetic counseling [50].

## Ethics Statement

The Ethics Committee on Human Research at People Hospital in the Ningxia Hui Autonomous Region accepted and examined this work (Reference Number: 20190909), which adhered to the Declaration of Helsinki. Each participant or their legal guardians provided their written informed permission before participation.

## Disclosure

All authors approved the final manuscript and its submission to this journal. Part of this research was previously shared as a preprint on Research Square [50]. The current manuscript has been substantially revised and expanded for formal peer‐reviewed publication.

## Conflicts of Interest

The authors declare no conflicts of interest.

## Author Contributions

Wan‐Yu Cheng and Wei‐Ning Rong wrote the main manuscript, Hui‐Ping Li made the protein–protein docking, Xiao‐Guang Wang, Rui Qi, and Xiao‐Long Qi collected case data and followed up with patients. Xun‐Lun Sheng and Wei Chi polished the article. All authors reviewed the manuscript. The co‐first authors are Wan‐Yu Cheng and Wei‐Ning Rong.

## Funding

This work was supported by the Key Research and Development Project of Ningxia Hui Autonomous Region (2020BEG03047, 2021BEG02045, and 2024BEG02017), the Training Project of the Scientific Innovation Commanding Talented Person in Ningxia Hui Autonomous Region (2020GKLRLX13), and the Major Achievement Transformation Project of Ningxia Hui Autonomous Region (2022CJE09011).

## Data Availability

The datasets generated and analyzed during the current study are available in the BankIt repository (https://www.ncbi.nlm.nih.gov/WebSub/) (ID: 2791933).
